# The role of GLI1 for 5-Fu resistance in colorectal cancer

**DOI:** 10.1186/s13578-017-0145-7

**Published:** 2017-04-13

**Authors:** Lining Zhang, Ruolan Song, Dongsheng Gu, Xiaoli Zhang, Beiqin Yu, Bingya Liu, Jingwu Xie

**Affiliations:** 1 Shanghai Key Laboratory of Gastric Neoplasms, Shanghai Institute of Digestive Surgery, Ruijin Hospital, Shanghai Jiao Tong University School of Medicine, Shanghai, 200025 China; 2 Departments of Pediatrics, Biochemistry and Molecular Biology, Pharmacology and Toxicology, The Wells Center for Pediatrics Research and IU Simon Cancer Center, Indiana University School of Medicine, Indianapolis, IN 46202 USA

**Keywords:** Colorectal cancer, Fluorouracil, GLI1, Hedgehog, EMT

## Abstract

Colorectal cancer is a leading cause of cancer-related mortality worldwide, with Fluorouracil (5-FU)-based chemotherapy as the major treatment for advanced disease. Many patients with advanced colorectal cancer eventually succumb to the disease despite some patients responded initially to chemotherapy. Thus, identifying molecular mechanisms responsible for chemotherapy resistance will help design novel strategies to treat colorectal cancer. In this study, we established an acquired 5-FU resistant cell line, LoVo-R, from LoVo cells. Through exome sequencing, we discovered that elevated GLI1 signaling axis is a major genetic alteration in the 5-FU resistant cells. Hh signaling, a pathway essential for embryonic development, is an important regulator for residual cancer cells. We demonstrated that knockdown of *GLI1* or *GLI2* sensitized LoVo-R cells to 5-FU treatment, reduced cell invasiveness. The relevance of our studies to colorectal cancer patients is reflected by our discovery that high expression of *GLI1* signaling molecules was associated with a high incidence of cancer relapse and a shorter survival in a larger cohort of colorectal cancer patients who underwent chemotherapy (containing 5-FU). Taken together, our data demonstrate the critical role of the GLI1 signaling axis for 5-FU resistance in colorectal cancer.

**Electronic supplementary material:**

The online version of this article (doi:10.1186/s13578-017-0145-7) contains supplementary material, which is available to authorized users.

## Background

The overall incidence of CRC has significantly declined in the last two decades, largely due to early screening and preventative measures in life styles. Currently, colorectal cancer (CRC) is still the leading cause of cancer-related deaths, with nearly 1.4 million cases a year and ~774,000 deaths worldwide [1].

Fluorouracil (FU)-based adjuvant chemotherapy has been used to treat CRC since 1990s. Adding oxaliplatin to FU has resulted in an approximate 20% relative risk reduction for disease-free survival. At present, the first line treatment of colorectal cancer includes mFOLFOX6 with or without targeted drugs bevacizumab or cetuximab [2, 3]. The mFOLFOX6 regimen contains leucovorin calcium (folinic acid), fluorouracil, and oxaliplatin. While some patients respond initially to chemotherapy, many advanced colorectal cancer patients eventually develop relapsed disease. Therefore, drug resistance is a major barrier to achieve effective gastric cancer treatment.

5-Fu, an analog of uracil with a fluorine atom substituted at the carbon-5 position of the pyrimidine ring in place of hydrogen, fulfills the expectations of biochemical, pharmacologic, and clinical activity of anticancer drugs. The 5-fluorinated pyrimidines have been widely used in the treatment of breast, gastric, colorectal, pancreatic cancers, and squamous cell carcinomas arising in the head and neck [4]. The primary mechanisms of action for 5-Fu include (1) incorporation of fluorouridine triphosphate into RNA to interfere with RNA synthesis and function; (2) inhibition of thymidylate synthase; (3) incorporation of fluorodeoxyuridine triphosphate and deoxyuridine triphosphate into DNA; and (4) genotoxic stress to trigger programmed cell death pathways.

Resistance to 5-FU in CRC is a major clinical problem. While there are a number of mechanisms reported to be responsible for drug resistance [5–9], activation of hedgehog (Hh), wnt and notch signaling pathways is quite appealing [10–12]. Like wnt and notch signaling, hedgehog signaling is an important regulator for embryonic development, tissue polarity, cell differentiation and cancer development [5, 9, 13–15]. Thus, specific inhibitors for these signaling pathways may be used to sensitize cancer cells to 5-FU treatment. However, the significance of Hh (or wnt and notch signaling) for 5-FU resistance in colorectal cancer has not been well established.

To elucidate the underlying mechanism for 5-FU resistance in CRC, we established an acquired resistant cell line, LoVo-R, through addition of increasing amount of 5-FU to the LoVo parental cells for 1 year. We compared gene expression profiles of LoVo-R cells with that of the parental LoVo cells using next generation sequencing. We discovered elevated expression of Gli1 as the major change in the 5-FU resistant LoVo-R cells. We demonstrated the significance of Gli transcription factors for 5-FU resistance. The relevance of our studies in this pair of cell lines was reflected in a large cohort of patients with colorectal cancer who underwent 5-FU-based chemotherapy.

## Results

### Characteristics of 5-Fu-resistant LoVo-R cell line

**Fig. 1:**
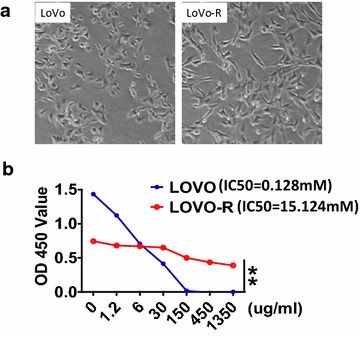
Characterization of 5-FU resistant LoVo-R cells. **a** Shows morphology of LoVo and LoVo-R cells. **b** Shows the IC50 dose of 5-FU calculated from measurement of cell viability in different concentrations of 5-FU (48 h). The *X-axis* is 5-FU concentration (μg/ml), and the *Y-axis* is O.D. values. Significant difference was indicated by *p < 0.05, **p < 0.005, or ***p < 0.0005

LoVo cells were cultured in medium containing stepwise increased concentrations of 5-Fu for 12 months to obtain LoVo-R cell line. There are a number of differences between the 5-FU resistant LoVo-R cells and the parental LoVo cells. Morphologically, we observed significant difference between LoVo and LoVo-R cells under microscope. Whereas LoVo-R cells have a spindle shape, LoVo cells are more epithelial cell-like (Fig. 1a). Second, we noticed that LoVo-R cells proliferate much slower than the parental cells. The doubling time for LoVo cells is ~48 h. In contrast, LoVo-R cells have a doubling time of ~96 h. By CCK8 assay, we found that the IC50 for 5-FU in LoVo-R cells is 1967.224 μg/ml (15.124 mM) (Fig. 1a). On the other hand, the IC50 for 5-FU in the parental LoVo cells is 16.6 μg/ml (0.128 mM). The resistant index is over 118 (=15.124/0.128), suggesting that LoVo-R is a true 5-FU resistant cell line.

### Gene expression analysis in LoVo-R and the parental LoVo cells

**Fig. 2:**
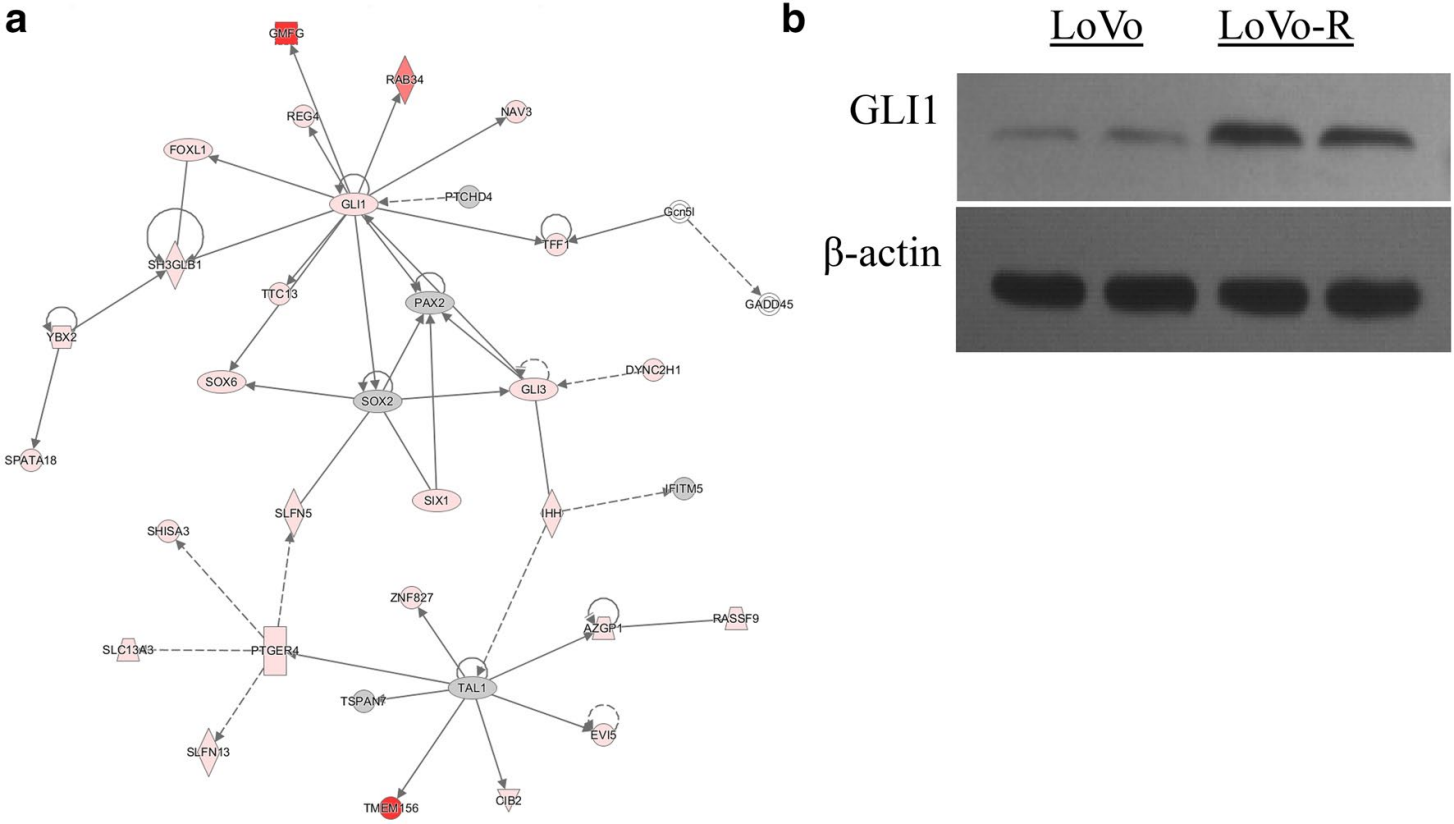
Up-regulated of GLI1 signaling axis in LoVo-R (in comparison with LoVo) cells. After next generation sequencing, we performed ingenuity pathway analysis (IPA). **a** Shows up-regulation of GLI1 and its signaling molecules, and the up-regulated genes are in *red*. **b** Detection of GLI1 protein in LoVo and LoVo-R cells. β-actin was used as the internal control

To understand the molecular basis responsible for drug resistance to 5-Fu in LoVo-R cells, we compared gene expression profiles between LoVo-R and LoVo cells using next generation sequencing. We had three biological repeats for each cell type, and we had 150-bp reads in both directions for the sequencing (see “Methods” for details). Overall, we observed gene expression changes in over 10,000 transcripts, with >7000 up-regulated and 4000 down-regulated in LoVo-R cells (Datasets submitted to GEO datasets, with an ID: 379155). Significant changes were listed in Additional file 1: Figures S1–S5. Pathway analyses using the IPA program indicate alterations in several signaling pathways, including growth factors (VEGF, MEK), EMT regulation and hedgehog signaling (Additional file 1 and Fig. 2a). Hedgehog signaling related regulator GLI1 is known to be vital in cancer biology [16, 17]. GLI1 is known to be significantly overexpressed in colorectal cancer cells [18]. As a major focus for our laboratory on Hh signaling, we proposed that GLI1 might be a vital factor of 5-Fu resistance in patients with colorectal cancer.

To confirm the sequencing data, we performed Western blotting analysis using GLI1 specific antibodies. We found that GLI1 protein was higher in LoVo-R cells in comparison with the parental LoVo cells (Fig. 1b).

### Functional significance of GLI1 for 5-FU resistance in colorectal cancer cells

**Fig. 3:**
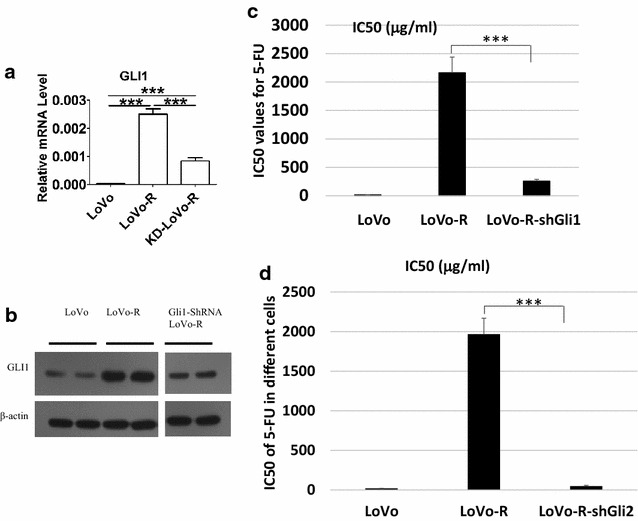
The effect of *GLI1/2* knockdown on 5-FU response in LoVo-R cells. **a** Real-time PCR detection of *GLI1* after *GLI1* shRNA expression. **b** Detection of GLI1 protein by Western blotting. **c** The effect of *GLI1*-ShRNAs (shown as shGLI1) on the IC50 of 5-FU (measured as shown in Fig. 1b). **d** Effects of GLI2 shRNAs (shown as shGLI2) on 5-FU response. Significant difference was indicated by *p < 0.05, **p < 0.005, or ***p < 0.0005

Considering the functional domains of GLI1, we designed three shRNAs to knock down GLI1: shRNA#1, shRNA#2 and shRNA#3. Lentiviruses expressing GLI1 shRNAs were used to infect LoVo-R cells. To test the change of LoVo-R cell line in 5-FU resistance after knocking down GLI1, we used CCK8 assay to detect cell viability in the presence or absence of 5-FU (Fig. 3). As expected, Gli1 shRNA expression sensitized LoVo-R cells to 5-FU treatment, with the IC50 around 280 μg/ml (2.154 mM), which was much lower to 1967.224 μg/ml (15.124 mM) in LoVo-R cells. However, the IC50 was not reduced to the level of the parental LoVo cells, which was 16.041 μg/ml (0.123 mM), indicating that there are other factors involved in regulation of 5-FU resistance.

**Fig. 4:**
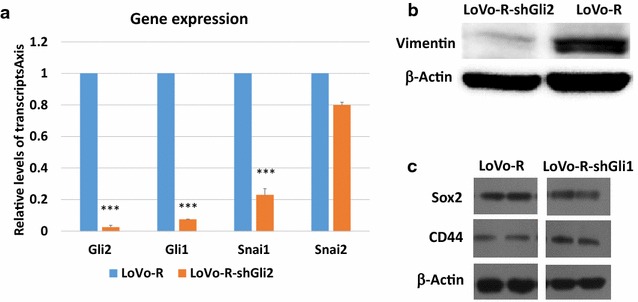
The effects of *GLI1 knockdown* on gene/protein expression. **a** Real-time analysis of *GLI1, GLI2, Snai1 and Snai2* in GLI2 shRNAs-expressing LoVo-R cells. Significant difference was indicated by ***p < 0.0005. **b** Effects of Gli2 shRNAs on vimentin expression. **c** The effect of GLI1 knockdown on Sox2 and CD44 proteins

It is known that GLI2 can regulate Gli1 expression. Although the *Gli2* expression was not much changed in LoVo-R cells, expression of Gli2 was detectable in both LoVo and LoVo-R cells. We tested whether Gli2 shRNAs affect GLI1 expression and 5-FU resistance in LoVo-R cells. We found that the IC50 for 5-FU in Gli2-shRNA-expressing LoVo-R cells (shown as shGli2) was 0.36 mM (the control LoVo-R with an IC50 for 5-FU of 15 mM), indicating significantly reduction of IC50 by *GLI2* shRNAs (p < 0.05) (Fig. 3d). As expected, we found reduced expression of both Gli1 and Gli2 in the Gli2 shRNA-expressing LoVo-R cells (Fig. 4a).

### Molecular mechanisms for GLI1-mediated 5-FU resistance in colorectal cancer cells

**Fig. 5:**
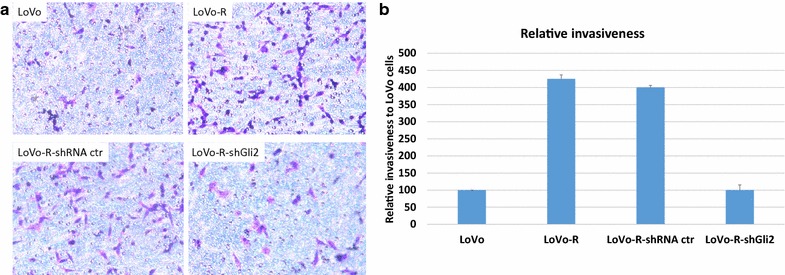
Cell invasiveness assay. Different cells were subjected to cell invasiveness as described in the methods, and invasive cells were visualized by blue in the staining (**a**). **b** Shows the summary from three independent experiments, with the value (number of invasive cells) from LoVo cells as 100

To determine the molecular mechanisms underlying Gli1-mediated 5-FU resistance in LoVo-R cells, we characterized LoVo-R cells with GLI1-shRNAs or GLI2-shRNAs for cell morphology, EMT and expression of cancer stem cell markers. Although *GLI1* and *GLI2* shRNAs resulted in reduction of IC50 for 5-FU, we did not observe significant changes in cell morphology (data not shown), indicating that GLI1 is not responsible for the morphological change in LoVo-R cells. Next, we examined EMT markers, and found that GLI1/2 knockdown reduced expression of vimentin and Snai1 (Fig. 4a, b), indicating that EMT regulation is a major mechanism by which GLI1 promotes drug resistance in LoVo-R cells. In addition, we also assessed expression of cancer stem cell markers, and found no significant changes in CD44, a commonly used marker for colorectal cancer stem cells [19–21] (Fig. 4c). No significant changes were observed in Sox2 after GLI1 shRNA expression (Fig. 4c). From these, we predict that EMT regulation seems to be the major function of GLI1 in LoVo-R cells. While LoVo-R cells have more EMT phenotypes (spindle shaped morphology, high vimentin and snai1 expression), knockdown of GLI1 and GLI2 reduced expression of snai1 and vimentin. It is known that EMT phenotypes is often associated with cell invasiveness [22]. We examined cell invasiveness using Boyden chambers, and found that GLI2 knockdown significantly reduced cell invasiveness (Fig. 5). We found that LoVo-R cells increased the relative cell invasiveness by nearly three times, and GLI2 shRNA expression reduced the invasiveness back to the basal level. We did not see significant changes in cell invasiveness after expression of control shRNAs (Fig. 5). These data indicate that expression of Gli1 is critical for 5-FU resistance and for cell invasiveness in LoVo-R cells.

### Relevance of the Gli1 signaling axis in human colorectal cancer

**Fig. 6:**
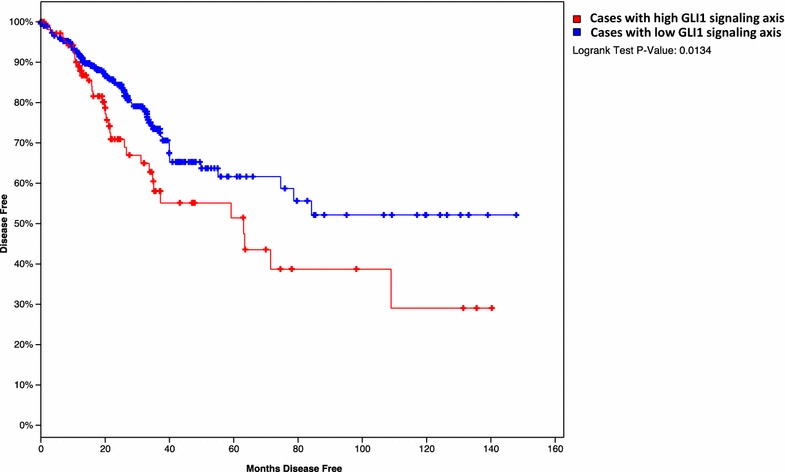
Kaplan–Meier Survival analysis comparing patients with high GLI1 signaling axis in the tumor with those patients with low GII1 signaling. The results here are in whole or part based upon data generated by the TCGA Research Network: http://cancergenome.nih.gov/

The relevance of our data to human colorectal cancer patients was reflected by analysis of cancer relapse in a large cohort of patients with colorectal cancer from the TCGA data sets (The results here are in whole or part based upon data generated by the TCGA Research Network: http://cancergenome.nih.gov/; http://www.cbioportal.org) [23, 24]. All patients underwent chemotherapy (all had 5-FU), which is the standard care for colorectal cancer patients. We correlated the high or low expression of *GLI1/GLI3/*pathway molecules in the tumor with cancer relapse (Additional file 2). We found that tumor recurrence occurred in 33% of the patients with tumors expressing high levels of GLI1 and the signaling molecules. In contrast, those patients with low *GLI1/GLI3* expression had only 19.5% of patients with relapse. The odd ratio for cancer relapse in high *GLI1/GLI3* expression is 1.69, indicating that high expression of *GLI1/GLI3* signaling molecules increases the risk of cancer relapse by 69% in colorectal cancer patients after chemotherapy. In consistent with cancer relapse, we also found that patients with high expression of GLI1/GLI3 and associated molecules in the primary tumors had worse patient survival (Fig. 6). Since all patients underwent chemotherapy (containing 5-FU), this correlation further support our hypothesis that high expression of *GLI1*/*GLI3* molecules in the tumor indicates poor outcomes from 5-FU associated chemotherapy treatment.

Taken all the data together, our study reveals a new mechanism for 5-FU resistance in colorectal cancer. We found activation of the GLI1 signaling axis in acquired resistant cancer cells to 5-FU treatment. Down-regulation of *GLI1* or *GLI2* sensitized cancer cells to 5-FU treatment. We believe that Gli1 mediates its resistance to 5-FU through direct regulation of EMT and cell invasiveness. The relevance of our data to colorectal cancer patients is reflected by the increasing risk of cancer relapse and poor treatment outcomes in patients with high *GLI1* expression in the primary tumor.

## Discussion

Our study has revealed a novel mechanism responsible for 5-FU based chemotherapy resistance and its link to cancer relapse. As a major contributor for cancer-related mortality, many colorectal cancer patients are diagnosed with advanced disease where the five-year survival rate is still low (12%) [25]. Chemotherapy with 5-FU has been the first line treatment option for advanced colorectal cancer, but many patients develop cancer relapse after initial treatment. Although the regulatory mechanisms for chemotherapy resistance in colorectal cancer have been reported, few studies have been linked the mechanisms to cancer relapse [18, 26–36]. Our results indicate that elevated expression of *GLI1* and the signaling molecules are an important mechanism for 5-FU resistance in colorectal cancer cells. We have data to show that knocking down *GLI1 or GLI2* will sensitize cancer cells to 5-FU treatment. More importantly, we have shown that high expression of *GLI1* and its signaling molecules is associated with an increasing risk of developing cancer relapse and a poor survival in colorectal cancer patients who underwent 5-FU based chemotherapy, indicating that our studies is relevant to the colorectal cancer patients. While the additional understanding of GLI1-mediated 5-FU resistance is still needed, we have shown that regulation of EMT is a major mechanism. Since inhibitors for GLI1 are already available, we predict that these novel reagents, together with chemotherapy, will improve the overall survival of gastric cancer patients.

It is unlikely that any one mechanism will be responsible for all the resistance. There are many reports on the molecular mechanisms responsible for resistance to cytotoxic chemotherapy or targeted therapy, particularly for the acquired resistance. In our studies, we also noticed many changes in the acquired resistant LoVo-R cells, including morphological change, cell proliferation change and gene expression changes (Figs. 1 and 2). While knockdown of *GLI1* and *GLI2* sensitized cancer cells to 5-FU, the IC50 was still higher than the parental cells. Thus, we believe that there are other mechanisms responsible for 5-FU resistance in LoVo-R cells, which will need further investigation.

Identifying GLI1 as a factor driving 5-FU resistance has significant clinical implications. First, our studies explained why SMO antagonists did not work in colorectal cancer treatment in previous clinical trials [37, 38]. We found that Shh expression was not significantly altered by 5-FU in LoVo-R cells. We further demonstrated and SMO antagonist BMS833923 was not able to sensitize cancer cells to 5-FU (data not shown here), suggesting that up-regulation of *GLI1* was not caused by canonical Hh signaling. This implies that the SMO antagonists, such as vismodegib [39], will not be effective in sensitizing colorectal cancer cells to chemotherapy, which was shown in the previous clinical trial. Second, our data indicate that specific inhibitors to GLI molecules may be more effective in sensitizing cancer cells to 5-FU based chemotherapy. Currently, there are several small molecules now available to target GLI1, such as GANT61 and arsenic trioxide [40–43].

## Conclusions

We report a novel mechanism by which colorectal cancer cells gain acquired fluorouracil (5-FU) resistance. We found that elevated GLI1 signaling axis is responsible for 5-FU resistance both in cultured cell lines and in relevant cancer patients. It is thus predicted that agents inhibiting GLI1 activity may be effective in sensitizing colorectal cancer cells to 5-FU-based chemotherapy.

## Methods

### LoVo cell line and cell culture

The human colorectal cancer cell line LoVo was purchased from ATCC. Cells were cultured in 90% RPMI 1640 medium (Gibco, 11875085) with 10% fetal bovine serum (FBS) (Gibco, 1009-141), 50 U/ml Penicillin and 50 μg/ml Streptomycin liquid (Gibco, 15070063) at 37 °C under 5% CO_2_ atmosphere (Thermo, HERAcell 240).

### Reagents and antibodies

5-fluorouracil reagent was purchased from Sigma (F6627). Powder was dissolved in dimethyl sulfoxide and sub-packed in 1.5 ml EP tubes, then stocked at −80 °C until use. Western blot antibodies against Gli1 (Cell signaling), CD44 (ABCam), E-Cadherin (ABCam), Snail (Cell Signaling) and Vimentin (Cell Signaling) were purchased from ABCam Inc. or Cell Signaling Technology Inc. The HRP-conjugated secondary antibodies were purchased from Santa Cruz Biotechnology.

### Establishment of 5-Fu-resistant LoVo-R cell line

5-Fu resistance colorectal cancer cell line LoVo-R was generated from parent LoVo cell line by exposed to gradient concentrations of 5-Fu. Briefly, LoVo cells were cultured in fresh medium without drugs for 24 h. Subsequently, medium was changed by adding 10 μM 5-Fu in complete medium. LoVo cells was exposed to 5-Fu for 48 h, then culture cells in fresh medium without 5-Fu for about a week. When cells reached 70% confluence, repeated above steps for several times until they were stable in 1× IC_50_. After that, cells were subjected into 2×, 3×…IC50. After 12 months’ selection, the 5-Fu-resistant cell line LoVo-R was used for this study.

### Determination of 50% cell growth inhibition (IC_50_) by CCK8 assay

Cells were adjusted to 5 × 10^4^/ml using complete medium, and inoculated to a 96-wells plate, with 100 μL cell suspension for each well. Five to eight wells were prepared for each sample. After cultured for 24 h, three treatment groups were used: group A with no cells for the background; group B without 5-Fu to calculate the basic metabolism; and group C with different concentrations of 5-Fu. Subsequently, 10 μg/ml CCK8 was added into each well for 1 h. Absorbance was detected by Microplate Reader (Thermo Scientific). A similar assay was also performed using Alarma blue assay [44].

### Cell migration assay

Migration of cells was performed using QCM™24-Well Colorimetric Migration Assay Kit (Millipore) according to the manufacturer’s instructions. Cells (1 × 10^5^) in 300 µl serum-free medium were added to the upper chambers and cultured for 48 h and 72 h. Non-migrating or non-invading cells were removed with cottons swabs, cells that migrated to the bottom of the membrane were stained, and counted under microscope and photographed. Three independent experiments were performed for each sample.

### Lentivirus vector construction and transfection

Based on Transcriptome Sequencing Database data, we designed three knock-down sites—shRNA#1, shRNA#2 and shRNA#3 for GLI1. The target sequences were as follows:

shRNA#1 (5′-CCGGTACATCAACTCCGGCCAATAGCTCGAGCTATTGGCCGGAGTTGATGTATTTTT); shRNA#2 (5′-CCGGCCTGATTATCTTCCTTCAGAACTCGAGTTCTGAAGGAAGATAATCAGGTTTTT);shRNA#3 (5′-CCGGGCTCAGCTTGTGTGTAATTATCTCGAGATAATTACACACAAGCTGAGCTTTTT). We purchased constructed GLI2 shRNAs and the control shRNAs from Sigma. Expression of shRNAs was donw with lentivirus-mediated infection. Stable clones were selected by continuous treatment with Puromycin (1.0 mg/ml; Gibco, New York, USA).

### Western blot analyses

Cells from 10 cm dishes were lysed in lysis buffer (1% NP-40) on ice for 30 min. Proteins were separated by SDS-PAGE, and then transferred to a onto a nitrocellulose membrane. Then the membrane was incubated in the first antibody followed by the secondary antibody. Protein bands were detected by using an enhanced chemiluminescence detection system. β-actin was used as the internal control.

### RNA preparation and real-time PCR

RNA was extracted from cells using TRIzol Reagent (Invitrogen) and 1 μg cDNA was synthesized from extracted total RNA using the PrimeScript™ RT-PCR Kit (Takara) according to the manufacture’s instruction. Quantitative PCR was carried out with the PrimeScript^®^ RT reagent Kit (Takara) on an ABI Prism 7900HT (Applied Biosystems, Foster City).

### Transcriptomic analysis using RNAseq

For transcriptomic analysis, total mRNA of LoVo and LoVo-R cells were extracted using TRIzol Reagent (Invitrogen). Equal amount of mRNA should be assured, then RNAs were used to construct the library. Sequencing was conducted using Illumina HiSeq-2000. Analyzing process was conducted as the normal bioinformatics analyzing method. The RNA abundance was evaluated by Reads per kilobases per million reads (RPKM).

### Statistical analyses

Analyses were performed using GraphPad Prism software for windows version 6 (Graphpad software, San Diego, CA). Student’s t tests were performed for statistical analysis with two groups. P value was calculated using unpaired ANOVA. Significance was discriminated as *p < 0.05, **p < 0.01, ***p < 0.001. A two-tailed value of p less than 0.05 was considered to be statistically significant. Odd ratio was calculated according to a previously described formula [45].

## Additional files

**Additional file 1.** Additional figures from IPA analyses.

**Additional file 2.** Additional Table.
