# Supplementary material for: The role of GLI1 for 5-Fu resistance in colorectal cancer
Source: Cell Biosci. 2017 Apr 13;7:17. doi: 10.1186/s13578-017-0145-7 (PMC5390459; doi:10.1186/s13578-017-0145-7)
Supplement: Supplementary file 1 — Additional file 1. Additional figures from IPA analyses. [file 13578_2017_145_MOESM1_ESM.pdf]

Supplementary information

Exome sequencing identifies a novel mechanism for 5-Fu resistance in colorectal cancer-

Lining Zhang[1], Ruolan Song[1, 2], Dongsheng Gu[2], Xiaoli Zhang[2], Beiqin Yu[1], Bingya Liu[1,*], Jingwu Xie[2, *]

[1]Shanghai Key Laboratory of Gastric Neoplasms, Shanghai Institute of Digestive Surgery, Ruijin Hospital, Shanghai Jiao Tong University School of Medicine, Shanghai 200025, China

[2]Departments of Pediatrics, Biochemistry and Molecular Biology, Pharmacology and Toxicology, The Wells Center for Pediatrics Research and IU Simon Cancer Center, Indiana University School of Medicine, Indianapolis, IN 46202, USA.

*Correspondence address. Jingwu Xie, E-mail: jinxie@iu.edu; Bingya Liu, Email: liubingya@sjtu.edu.cn

Email addresses for all authors: Lining Zhang- zln629@163.com; Ruolan Song- ruolsong@iu.edu; Dongsheng Gu- donggu@iu.edu; Xiaoli Zhang- zhang86@iu.edu; Beiqin Yu- yubeiqin@126.com; Bingya Liu- liubingya@sjtu.edu.cn; Jingwu Xie- jinxie@iu.edu

Supplementary figures from IPA analyses

Supplementary figure 1- Signaling networks

Network 2 : LoVo genes-20160714new-list - 2016-07-13 09:23 AM : LoVo genes-20160714new-list : LoVo genes-20160714new-list - 2016-07-13 09:23 AM

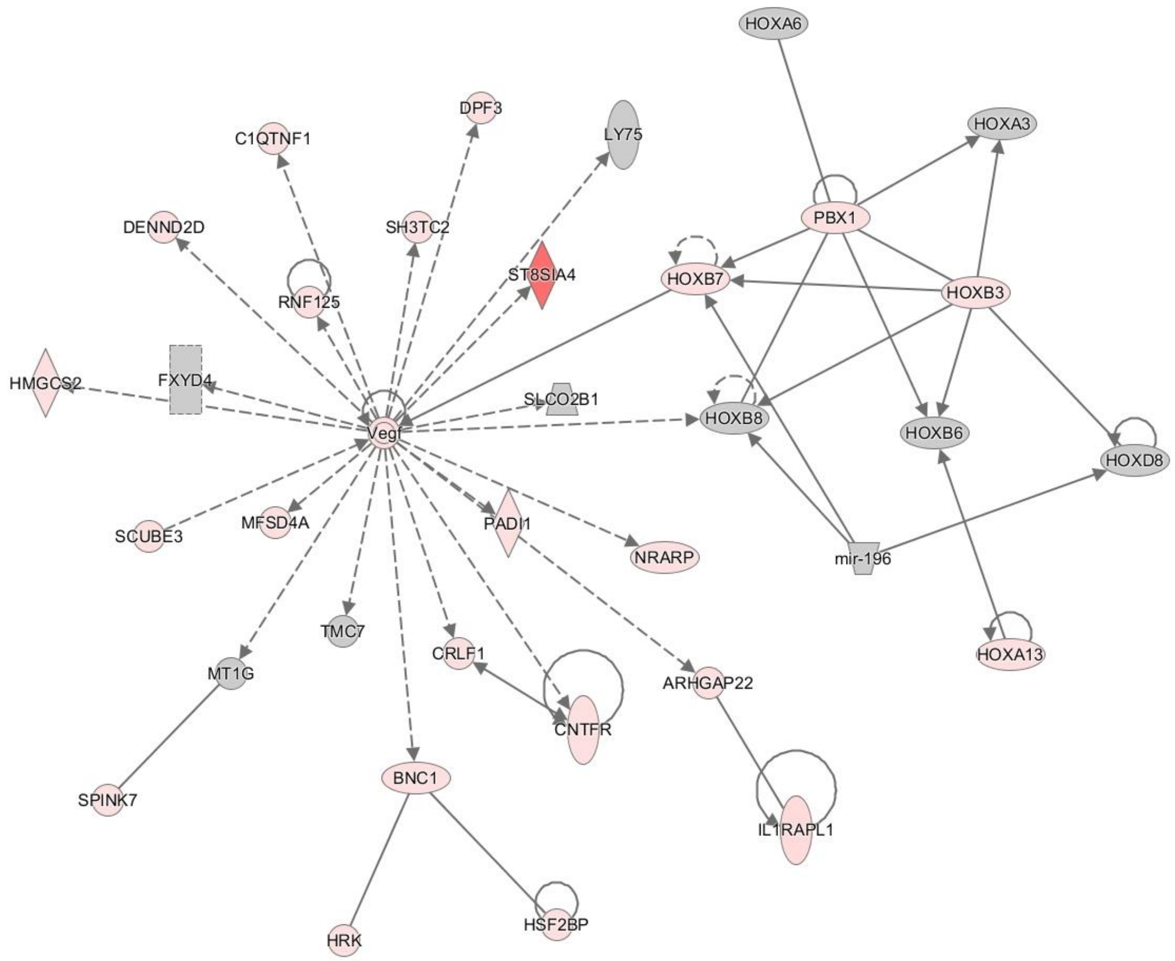

© 2000-2016 QIAGEN. All rights reserved.

Supplementary figure 2- Signaling networks

Network 9 : LoVo genes-20160714new-list - 2016-07-13 09:23 AM : LoVo genes-20160714new-list : LoVo genes-20160714new-list - 2016-07-13 09:23 AM

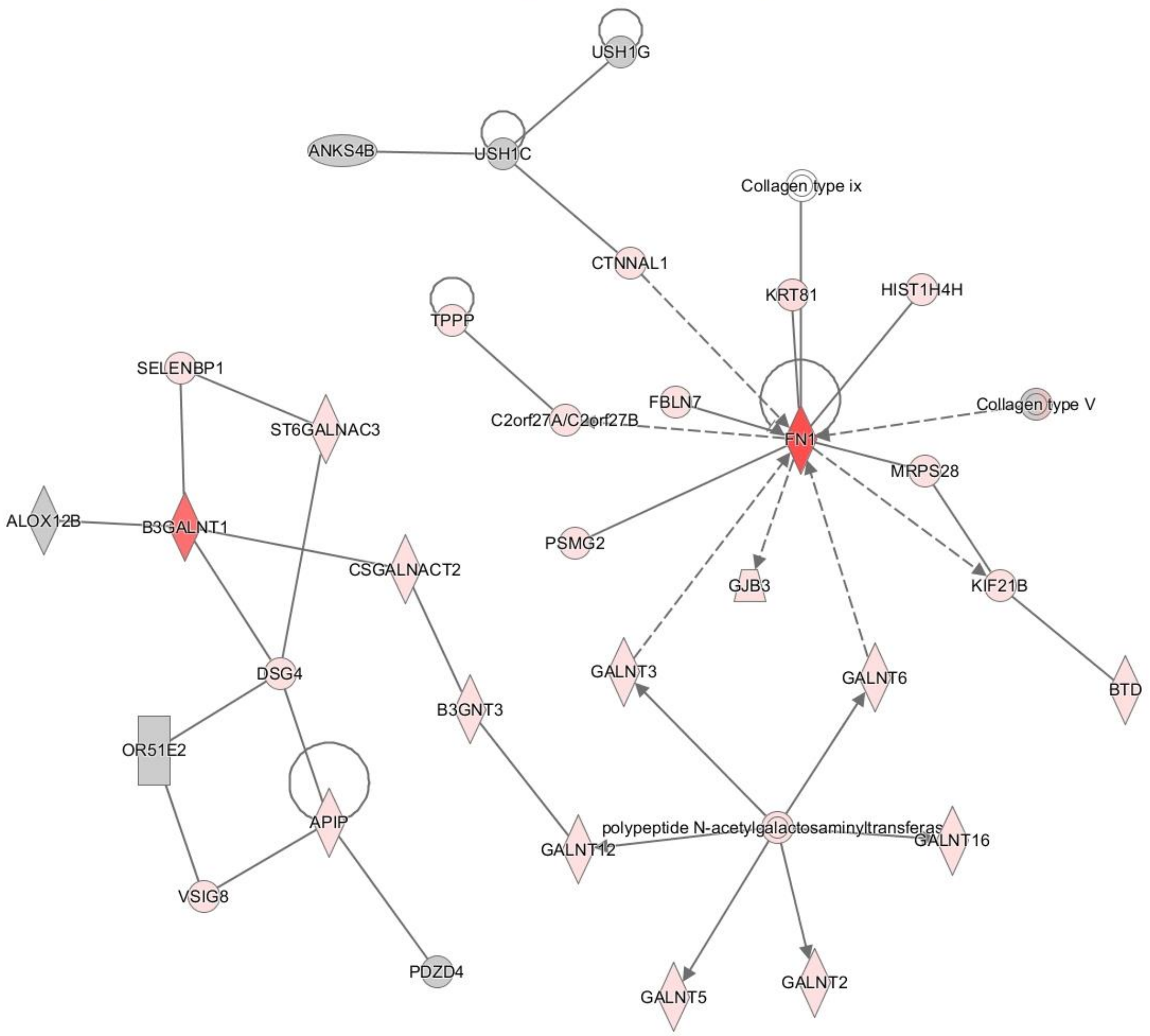

© 2000-2016 QIAGEN. All rights reserved.

Supplementary figure 3- Signaling networks

Network 17 : LoVo genes-20160714new-list - 2016-07-13 09:23 AM : LoVo genes-20160714new-list : LoVo genes-20160714new-list - 2016-07-13 09:23 AM

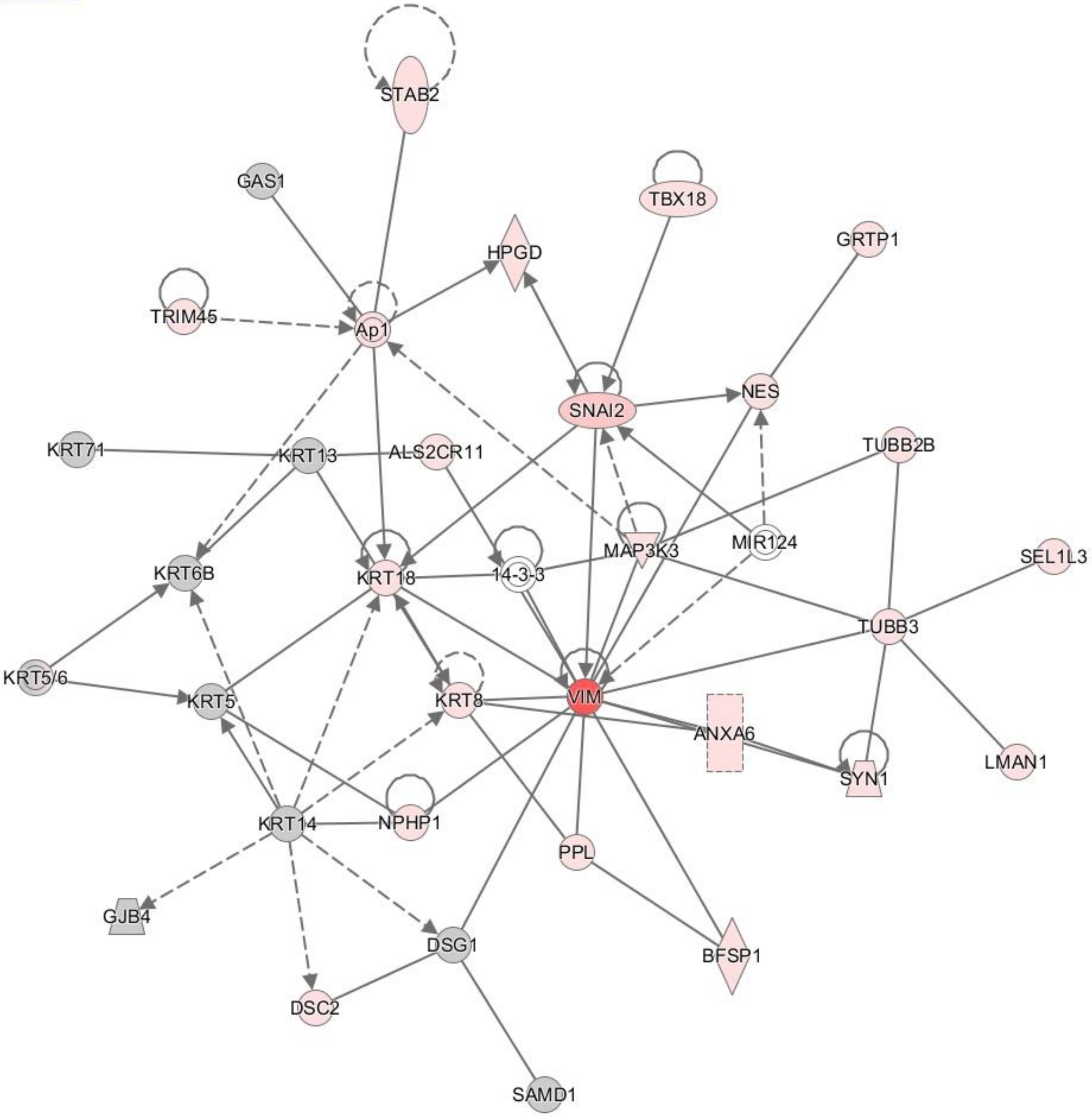

© 2000-2016 QIAGEN. All rights reserved.

Supplementary figure 4- Signaling networks

Network 20 : LoVo genes-20160714new-list - 2016-07-13 09:23 AM : LoVo genes-20160714new-list : LoVo genes-20160714new-list - 2016-07-13 09:23 AM

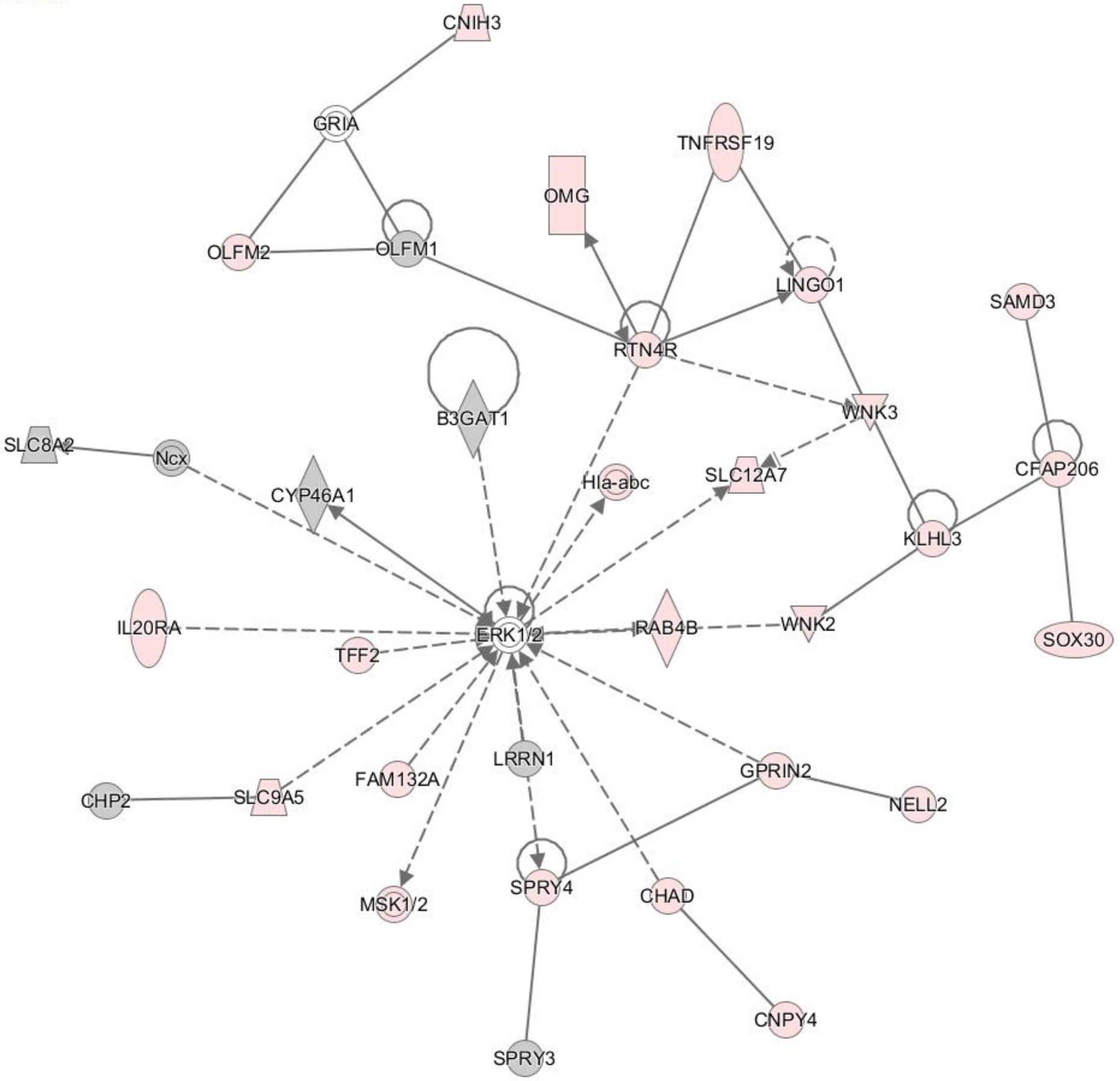

© 2000-2016 QIAGEN. All rights reserved.

Supplementary figure 5- Signaling networks

Network 23 : LoVo genes-20160714new-list - 2016-07-13 09:23 AM : LoVo genes-20160714new-list : LoVo genes-20160714new-list - 201 -07-13 09:23 AM

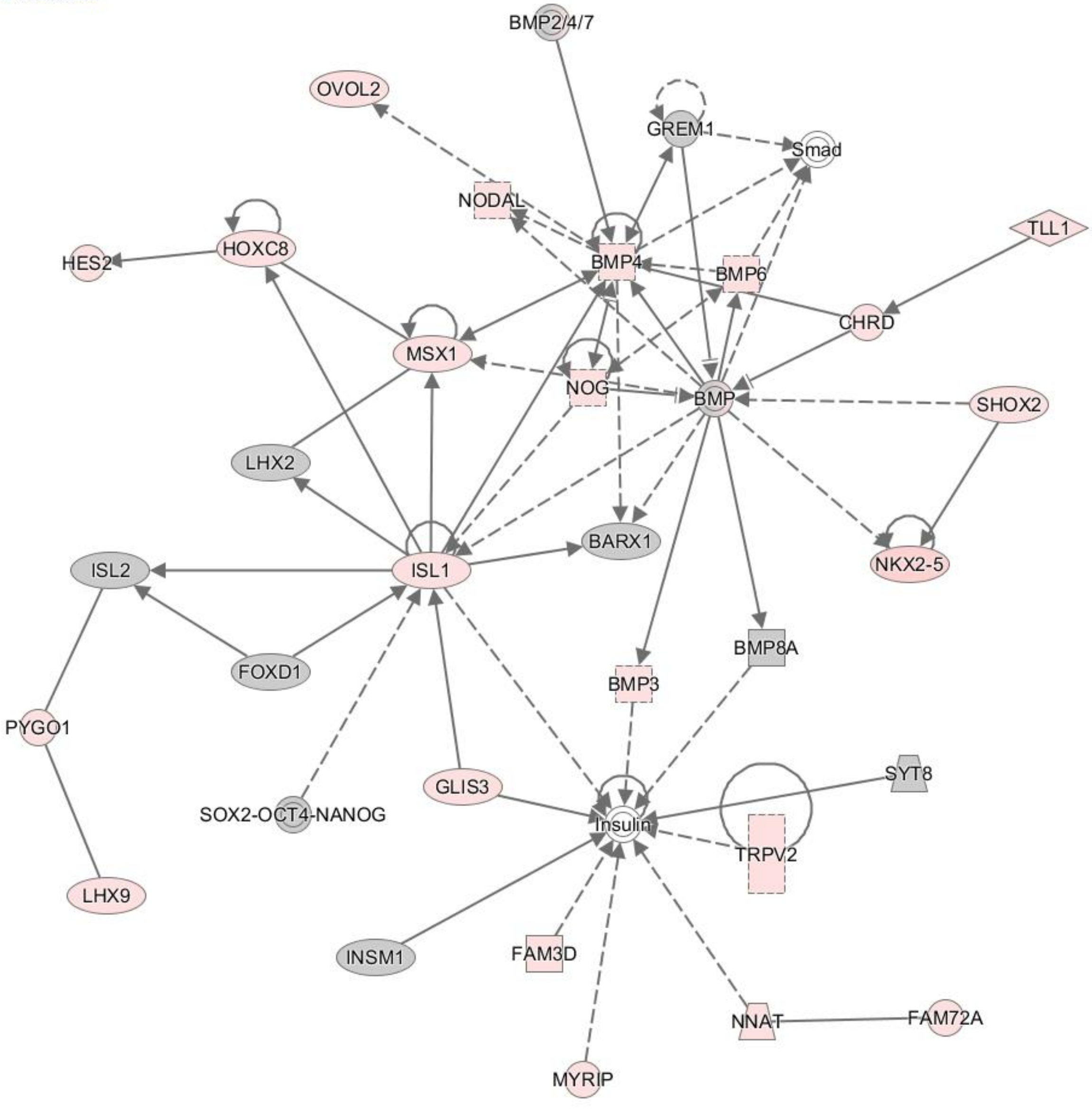

© 2000-2016 QIAGEN. All rights reserved.
