# Supplementary material for: The role of GLI1 for 5-Fu resistance in colorectal cancer
Source: Cell Biosci. 2017 Apr 13;7:17. doi: 10.1186/s13578-017-0145-7 (PMC5390459; doi:10.1186/s13578-017-0145-7)
Supplement: Supplementary file 2 — Additional file 2. Additional Table. [file 13578_2017_145_MOESM2_ESM.pdf]

<html><head></head><body><pre style="word-wrap: break-word; white-space: pre-wrap;">Disease Free Survival Kaplan-Meier Estimate

Cases with Alteration(s) in Query Gene(s)

Case ID	Number at Risk	Status	Survival Rate	Time (months)
TCGA-AA-3712	108	censored	1	0
TCGA-AY-6196	107	censored	1	0.2
TCGA-AM-5821	106	censored	1	0.92
TCGA-CM-5862	105	relapsed	0.9904761904761905	1.02
TCGA-F4-6704	104	censored	0.9904761904761905	1.54
TCGA-CK-6748	103	censored	0.9904761904761905	2
TCGA-F5-6813	102	relapsed	0.9807656395891691	2.14
TCGA-NH-A6GA	101	relapsed	0.9710550887021476	3.48
TCGA-4N-A93T	100	censored	0.9710550887021476	4.8
TCGA-CI-6619	99	censored	0.9710550887021476	6.04
TCGA-CA-6718	98	relapsed	0.9611463633072277	6.8
TCGA-5M-AAT6	97	relapsed	0.9512376379123079	7.19
TCGA-EI-7004	96	censored	0.9512376379123079	8.44
TCGA-A6-A566	95	relapsed	0.9412246101448098	8.44
TCGA-EI-6882	94	censored	0.9412246101448098	8.61
TCGA-D5-6927	93	censored	0.9412246101448098	9.43
TCGA-CM-6679	92	censored	0.9412246101448098	10.05
TCGA-AA-A02K	91	relapsed	0.9308814825608009	10.05
TCGA-F4-6460	90	relapsed	0.920538354976792	10.28
TCGA-DM-A288	89	relapsed	0.9101952273927831	10.51
TCGA-AD-6964	88	relapsed	0.8998520998087742	10.68
TCGA-CM-6172	87	censored	0.8998520998087742	11.01
TCGA-CM-5861	86	relapsed	0.8893887032993698	11.01
TCGA-D5-6928	85	censored	0.8893887032993698	11.63
TCGA-DC-6154	84	censored	0.8893887032993698	11.99
TCGA-CM-6162	83	censored	0.8893887032993698	11.99
TCGA-F5-6702	82	relapsed	0.878542499600597	12.09
TCGA-CA-6716	81	censored	0.878542499600597	12.19
TCGA-CA-5255	80	censored	0.878542499600597	12.35
TCGA-CA-5796	79	censored	0.878542499600597	12.39
TCGA-D5-6923	78	censored	0.878542499600597	12.42
TCGA-F5-6864	77	censored	0.878542499600597	12.45
TCGA-DT-5265	76	censored	0.878542499600597	12.61
TCGA-D5-6529	75	relapsed	0.8668285996059224	12.68
TCGA-NH-A6GC	74	censored	0.8668285996059224	12.78
TCGA-CM-6674	73	censored	0.8668285996059224	12.94
TCGA-D5-6930	72	censored	0.8668285996059224	13.34
TCGA-DC-6155	71	censored	0.8668285996059224	13.96
TCGA-CM-6171	70	censored	0.8668285996059224	14.03
TCGA-CM-6163	69	censored	0.8668285996059224	14.03
TCGA-A6-A565	68	relapsed	0.854081120199953	14.42
TCGA-CM-6167	67	censored	0.854081120199953	14.98
TCGA-CK-4951	66	relapsed	0.8411404971666203	15.77
TCGA-DM-A28H	65	relapsed	0.8281998741332878	15.9
TCGA-D5-6536	64	relapsed	0.8152592510999551	16.2
TCGA-EI-6514	63	censored	0.8152592510999551	16.29
TCGA-NH-A8F7	62	censored	0.8152592510999551	17.84
TCGA-AY-A69D	61	censored	0.8152592510999551	17.84
TCGA-NH-A50T	60	censored	0.8152592510999551	18.17
TCGA-AA-3713	59	censored	0.8152592510999551	19.02
TCGA-D5-5539	58	relapsed	0.8012030571154731	19.02
TCGA-AY-A71X	57	censored	0.8012030571154731	19.32
TCGA-A6-6781	56	censored	0.8012030571154731	19.65
TCGA-G4-6314	55	relapsed	0.7866357288042827	19.88
TCGA-EI-6507	54	censored	0.7866357288042827	19.94
TCGA-AF-6655	53	censored	0.7866357288042827	20.01
TCGA-AF-6136	52	relapsed	0.7715081186349695	20.01
TCGA-DM-A0XD	51	relapsed	0.7563805084656563	20.27
TCGA-EI-6506	50	censored	0.7563805084656563	20.53
TCGA-A6-5664	49	relapsed	0.7409441715581939	21.12
TCGA-BM-6198	48	censored	0.7409441715581939	21.22
TCGA-AY-6197	47	censored	0.7409441715581939	21.42
TCGA-A6-6142	46	relapsed	0.7248366895677985	21.48
TCGA-A6-5662	45	relapsed	0.7087292075774029	21.71
TCGA-A6-6651	44	censored	0.7087292075774029	21.75

TCGA-QL-A97D	43	censored	0.7087292075774029	21.88
TCGA-CM-6166	42	censored	0.7087292075774029	21.98
TCGA-G4-6321	41	censored	0.7087292075774029	22.08
TCGA-CM-5348	40	censored	0.7087292075774029	22.96
TCGA-A6-6654	39	censored	0.7087292075774029	23.85
TCGA-A6-6649	38	censored	0.7087292075774029	24.15
TCGA-A6-6652	37	censored	0.7087292075774029	24.67
TCGA-AA-A01X	36	relapsed	0.6890422851446972	25.99
TCGA-5M-AATE	35	relapsed	0.6693553627119916	26.61
TCGA-AH-6644	34	censored	0.6693553627119916	27.53
TCGA-A6-2685	33	relapsed	0.6490718668722343	31.14
TCGA-CM-5860	32	censored	0.6490718668722343	32
TCGA-F5-6811	31	censored	0.6490718668722343	32.16
TCGA-AH-6643	30	relapsed	0.6274361379764931	33.71
TCGA-AG-3591	29	censored	0.6274361379764931	34
TCGA-F4-6805	28	censored	0.6274361379764931	34.4
TCGA-F5-6571	27	relapsed	0.6041977624958822	34.79
TCGA-AA-3502	26	censored	0.6041977624958822	34.99
TCGA-A6-A567	25	relapsed	0.5800298519960468	35.12
TCGA-F4-6856	24	censored	0.5800298519960468	35.28
TCGA-F4-6569	23	censored	0.5800298519960468	35.71
TCGA-AA-3685	22	censored	0.5800298519960468	37.02
TCGA-F5-6814	21	censored	0.5800298519960468	37.16
TCGA-F4-6806	20	relapsed	0.5510283593962445	37.19
TCGA-D5-6534	19	censored	0.5510283593962445	43.23
TCGA-AA-3675	18	censored	0.5510283593962445	47.01
TCGA-F4-6855	17	censored	0.5510283593962445	47.37
TCGA-F4-6703	16	censored	0.5510283593962445	47.83
TCGA-SS-A7HO	15	relapsed	0.5142931354364949	59.17
TCGA-AA-3509	14	censored	0.5142931354364949	62.91
TCGA-DM-A1D4	13	relapsed	0.47473212501830303	63.07
TCGA-DM-A28C	12	relapsed	0.4351711146001111	63.37
TCGA-G4-6310	11	censored	0.4351711146001111	63.57
TCGA-WS-AB45	10	censored	0.4351711146001111	69.97
TCGA-G4-6309	9	relapsed	0.38681876853343206	71.48
TCGA-G4-6299	8	censored	0.38681876853343206	74.51
TCGA-AA-3660	7	censored	0.38681876853343206	78.02
TCGA-CL-5917	6	censored	0.38681876853343206	78.06
TCGA-DM-A28K	5	censored	0.38681876853343206	98.16
TCGA-DY-A1DE	4	relapsed	0.29011407640007403	108.94
TCGA-DM-A1HA	3	censored	0.29011407640007403	131.41
TCGA-DM-A1HB	2	censored	0.29011407640007403	135.55
TCGA-DM-A1D9	1	censored	0.29011407640007403	140.28

Cases without Alteration(s) in Query Gene(s)

Case ID	Number at Risk	Status	Survival Rate	Time (months)
TCGA-AG-4021	434	relapsed	0.9976958525345622	0
TCGA-AA-3814	433	censored	0.9976958525345622	0
TCGA-AA-3527	432	censored	0.9976958525345622	0
TCGA-AA-3872	431	censored	0.9976958525345622	0
TCGA-AG-A01L	430	censored	0.9976958525345622	0
TCGA-AG-A01Y	429	censored	0.9976958525345622	0
TCGA-AG-A01W	428	censored	0.9976958525345622	0
TCGA-AA-3984	427	censored	0.9976958525345622	0
TCGA-AA-3684	426	censored	0.9976958525345622	0
TCGA-AG-3601	425	censored	0.9976958525345622	0
TCGA-AG-3602	424	censored	0.9976958525345622	0
TCGA-CM-6678	423	relapsed	0.9953372334978374	0
TCGA-AA-3695	422	censored	0.9953372334978374	0
TCGA-AA-3693	421	censored	0.9953372334978374	0
TCGA-CK-5915	420	censored	0.9953372334978374	0
TCGA-AD-A5EJ	419	censored	0.9953372334978374	0
TCGA-AA-A022	418	censored	0.9953372334978374	0
TCGA-AM-5820	417	censored	0.9953372334978374	0.46
TCGA-F4-6854	416	censored	0.9953372334978374	0.53
TCGA-CK-4947	415	relapsed	0.9929388305255535	0.69
TCGA-AA-3672	414	censored	0.9929388305255535	0.92
TCGA-AA-A02O	413	censored	0.9929388305255535	0.92
TCGA-DC-6156	412	relapsed	0.9905287848203944	0.99
TCGA-AA-3856	411	censored	0.9905287848203944	0.99
TCGA-AA-3543	410	censored	0.9905287848203944	0.99
[illegible]	[illegible]	[illegible]	[illegible]	[illegible]

TCGA-AG-3727	409	censored	0.9905287848203944	0.99
TCGA-AG-3605	408	censored	0.9905287848203944	0.99
TCGA-AG-3742	407	censored	0.9905287848203944	0.99
TCGA-AG-3878	406	censored	0.9905287848203944	0.99
TCGA-AA-A00R	405	censored	0.9905287848203944	0.99
TCGA-AG-4007	404	censored	0.9905287848203944	1.02
TCGA-AA-3821	403	censored	0.9905287848203944	1.02
TCGA-AA-3514	402	censored	0.9905287848203944	1.02
TCGA-AG-A01J	401	censored	0.9905287848203944	1.02
TCGA-AA-3518	400	censored	0.9905287848203944	1.02
TCGA-AG-A020	399	censored	0.9905287848203944	1.02
TCGA-AA-3496	398	censored	0.9905287848203944	1.02
TCGA-AA-3494	397	censored	0.9905287848203944	1.02
TCGA-AG-3586	396	censored	0.9905287848203944	1.02
TCGA-AG-3883	395	censored	0.9905287848203944	1.02
TCGA-AA-A01Q	394	censored	0.9905287848203944	1.02
TCGA-AA-A01S	393	censored	0.9905287848203944	1.02
TCGA-AA-A01V	392	censored	0.9905287848203944	1.02
TCGA-AG-3896	391	censored	0.9905287848203944	1.02
TCGA-AA-3511	390	relapsed	0.987988967423419	1.94
TCGA-AA-3966	389	censored	0.987988967423419	2
TCGA-T9-A92H	388	relapsed	0.9854426041053175	2.66
TCGA-EI-6509	387	relapsed	0.9828962407872159	2.96
TCGA-CI-6620	386	relapsed	0.9803498774691143	2.96
TCGA-AA-3555	385	relapsed	0.9778035141510127	2.99
TCGA-AA-3558	384	relapsed	0.9752571508329112	3.02
TCGA-G5-6233	383	relapsed	0.9727107875148095	3.02
TCGA-EF-5830	382	censored	0.9727107875148095	3.48
TCGA-AA-3552	381	relapsed	0.970157740828419	4.01
TCGA-AG-A00Y	380	relapsed	0.9676046941420284	4.04
TCGA-D5-6537	379	relapsed	0.9650516474556379	4.04
TCGA-EF-5831	378	censored	0.9650516474556379	4.17
TCGA-AA-3715	377	relapsed	0.9624918287621216	5.03
TCGA-DM-A1D7	376	relapsed	0.9599320100686053	5.06
TCGA-AA-3681	375	censored	0.9599320100686053	5.98
TCGA-AG-A00C	374	censored	0.9599320100686053	6.01
TCGA-AA-A01D	373	relapsed	0.9573584658056867	6.01
TCGA-AA-3662	372	censored	0.9573584658056867	6.04
TCGA-AG-3600	371	censored	0.9573584658056867	6.04
TCGA-AY-4070	370	relapsed	0.9547710104926984	6.11
TCGA-F5-6863	369	relapsed	0.9521835551797101	6.27
TCGA-AA-3663	368	censored	0.9521835551797101	6.96
TCGA-AG-3581	367	censored	0.9521835551797101	7.06
TCGA-CL-5918	366	censored	0.9521835551797101	7.16
TCGA-D5-6898	365	censored	0.9521835551797101	7.52
TCGA-AG-3726	364	censored	0.9521835551797101	7.98
TCGA-CM-4750	363	censored	0.9521835551797101	8.02
TCGA-AG-3580	362	censored	0.9521835551797101	8.02
TCGA-AA-3525	361	censored	0.9521835551797101	8.05
TCGA-G4-6295	360	censored	0.9521835551797101	8.34
TCGA-A6-6141	359	censored	0.9521835551797101	8.38
TCGA-RU-A8FL	358	relapsed	0.9495238245786495	8.41
TCGA-D5-6926	357	censored	0.9495238245786495	9.03
TCGA-AA-3519	356	censored	0.9495238245786495	9.07
TCGA-NH-A50U	355	relapsed	0.9468491095798364	9.07
TCGA-AG-A016	354	censored	0.9468491095798364	9.07
TCGA-F4-6461	353	relapsed	0.9441668174847093	9.46
TCGA-G4-6298	352	relapsed	0.9414845253895824	9.56
TCGA-CK-5916	351	relapsed	0.9388022332944554	9.59
TCGA-A6-A5ZU	350	censored	0.9388022332944554	9.63
TCGA-EI-6885	349	relapsed	0.9361122555486261	9.69
TCGA-A6-2683	348	relapsed	0.9334222778027967	9.86
TCGA-AZ-5403	347	relapsed	0.9307323000569673	9.99
TCGA-D5-6922	346	censored	0.9307323000569673	10.12
TCGA-D5-7000	345	censored	0.9307323000569673	10.25
TCGA-AF-2692	344	relapsed	0.9280266829056388	10.74
TCGA-EI-6884	343	censored	0.9280266829056388	10.78
TCGA-CM-6676	342	censored	0.9280266829056388	11.07
TCGA-CM-6677	341	censored	0.9280266829056388	11.07
TCGA-CM-6675	340	relapsed	0.9252971926617988	11.07
TCGA-AD-6888	339	relapsed	0.9225677024179586	11.27
TCGA-D5-6932	338	censored	0.9225677024179586	11.37

TCGA-D5-6932	338	censored	0.9225677024179586	11.57
TCGA-EI-6883	337	censored	0.9225677024179586	11.5
TCGA-CA-6719	336	relapsed	0.9198219652083813	11.63
TCGA-AF-A56N	335	censored	0.9198219652083813	11.83
TCGA-EI-7002	334	censored	0.9198219652083813	11.96
TCGA-A6-4105	333	relapsed	0.9170597370846324	11.96
TCGA-AA-3844	332	relapsed	0.9142975089608835	11.99
TCGA-D5-6931	331	censored	0.9142975089608835	11.99
TCGA-AG-3575	330	censored	0.9142975089608835	11.99
TCGA-AA-A01G	329	censored	0.9142975089608835	11.99
TCGA-AG-3599	328	censored	0.9142975089608835	12.02
TCGA-CM-6680	327	censored	0.9142975089608835	12.02
TCGA-AD-5900	326	censored	0.9142975089608835	12.16
TCGA-D5-6920	325	censored	0.9142975089608835	12.39
TCGA-CA-5256	324	censored	0.9142975089608835	12.45
TCGA-D5-6539	323	censored	0.9142975089608835	12.48
TCGA-A6-2682	322	relapsed	0.911458075703241	12.52
TCGA-CA-6715	321	censored	0.911458075703241	12.58
TCGA-CA-5797	320	censored	0.911458075703241	12.58
TCGA-4T-AA8H	319	censored	0.911458075703241	12.65
TCGA-CA-5254	318	censored	0.911458075703241	12.68
TCGA-CA-6717	317	censored	0.911458075703241	12.75
TCGA-NH-A6GC	316	censored	0.911458075703241	12.78
TCGA-AA-3867	315	relapsed	0.9085645580025958	12.94
TCGA-AA-3939	314	censored	0.9085645580025958	12.98
TCGA-AA-3542	313	censored	0.9085645580025958	12.98
TCGA-CM-6168	312	censored	0.9085645580025958	12.98
TCGA-AA-3842	311	relapsed	0.9056431285556422	13.01
TCGA-AY-A54L	310	relapsed	0.9027216991086886	13.01
TCGA-CM-4752	309	censored	0.9027216991086886	13.01
TCGA-AA-3692	308	relapsed	0.8997907845011929	13.01
TCGA-CM-6169	307	censored	0.8997907845011929	13.01
TCGA-AG-3892	306	censored	0.8997907845011929	13.01
TCGA-AA-3973	305	censored	0.8997907845011929	13.04
TCGA-D5-6929	304	censored	0.8997907845011929	13.4
TCGA-AZ-4684	303	relapsed	0.8968211779516839	13.5
TCGA-G4-6323	302	censored	0.8968211779516839	13.76
TCGA-CI-6621	301	censored	0.8968211779516839	13.76
TCGA-AA-3561	300	censored	0.8968211779516839	13.93
TCGA-AG-A008	299	censored	0.8968211779516839	13.93
TCGA-AG-3611	298	censored	0.8968211779516839	13.93
TCGA-AA-A004	297	censored	0.8968211779516839	13.93
TCGA-AA-3544	296	censored	0.8968211779516839	13.99
TCGA-AA-3667	295	censored	0.8968211779516839	13.99
TCGA-AG-3894	294	censored	0.8968211779516839	13.99
TCGA-AG-4005	293	censored	0.8968211779516839	14.03
TCGA-D5-6924	292	censored	0.8968211779516839	14.29
TCGA-AU-3779	291	censored	0.8968211779516839	14.49
TCGA-AA-A00W	290	censored	0.8968211779516839	14.98
TCGA-CM-6170	289	censored	0.8968211779516839	15.01
TCGA-CM-5864	288	censored	0.8968211779516839	15.01
TCGA-CM-5863	287	censored	0.8968211779516839	15.01
TCGA-AA-3679	286	censored	0.8968211779516839	15.01
TCGA-CM-6161	285	censored	0.8968211779516839	15.01
TCGA-AA-A01C	284	censored	0.8968211779516839	15.01
TCGA-AA-A017	283	censored	0.8968211779516839	15.01
TCGA-D5-6535	282	censored	0.8968211779516839	15.11
TCGA-AF-6672	281	relapsed	0.8936296435105747	15.14
TCGA-AD-6901	280	relapsed	0.8904381090694655	15.37
TCGA-D5-6541	279	censored	0.8904381090694655	15.57
TCGA-CK-4952	278	censored	0.8904381090694655	15.6
TCGA-NH-A6GB	277	censored	0.8904381090694655	15.64
TCGA-EI-6511	276	censored	0.8904381090694655	15.83
TCGA-AA-3833	275	censored	0.8904381090694655	15.93
TCGA-AG-A014	274	censored	0.8904381090694655	15.93
TCGA-AG-3608	273	censored	0.8904381090694655	15.93
TCGA-CM-6165	272	censored	0.8904381090694655	16.03
TCGA-AA-3971	271	censored	0.8904381090694655	16.06
TCGA-D5-6540	270	censored	0.8904381090694655	16.13
TCGA-EI-6513	269	censored	0.8904381090694655	16.33
TCGA-EI-6881	268	censored	0.8904381090694655	16.39
TCGA-AD-A5EK	267	censored	0.8904381090694655	16.43

[illegible]	[illegible]	[illegible]	[illegible]	[illegible]
TCGA-G4-6294	266	relapsed	0.8870905973812344	16.98
TCGA-AG-4008	265	censored	0.8870905973812344	17.02
TCGA-AA-3846	264	censored	0.8870905973812344	17.02
TCGA-AA-3866	263	censored	0.8870905973812344	17.02
TCGA-CM-5868	262	censored	0.8870905973812344	17.02
TCGA-AG-A015	261	relapsed	0.883691782831881	17.02
TCGA-AG-3890	260	censored	0.883691782831881	17.02
TCGA-AA-A00U	259	censored	0.883691782831881	17.02
TCGA-D5-6538	258	censored	0.883691782831881	17.12
TCGA-A6-5665	257	relapsed	0.8802532934045196	17.38
TCGA-EI-6917	256	censored	0.8802532934045196	17.44
TCGA-AH-6549	255	censored	0.8802532934045196	17.48
TCGA-EI-6512	254	censored	0.8802532934045196	17.67
TCGA-D5-6531	253	censored	0.8802532934045196	17.74
TCGA-AY-6386	252	censored	0.8802532934045196	17.81
TCGA-NH-A8F7	251	censored	0.8802532934045196	17.84
TCGA-AA-3554	250	censored	0.8802532934045196	17.94
TCGA-AG-3885	249	censored	0.8802532934045196	17.94
TCGA-AA-3831	248	censored	0.8802532934045196	17.97
TCGA-AG-3891	247	censored	0.8802532934045196	18
TCGA-AA-3875	246	censored	0.8802532934045196	18.04
TCGA-AA-A00J	245	censored	0.8802532934045196	18.04
TCGA-AA-A00K	244	censored	0.8802532934045196	18.04
TCGA-D5-6532	243	censored	0.8802532934045196	18.23
TCGA-EI-6510	242	censored	0.8802532934045196	18.27
TCGA-AY-A8YK	241	censored	0.8802532934045196	18.82
TCGA-AG-A02G	240	relapsed	0.8765855713486674	18.99
TCGA-AA-3688	239	censored	0.8765855713486674	18.99
TCGA-AA-A00D	238	censored	0.8765855713486674	18.99
TCGA-AG-3881	237	censored	0.8765855713486674	19.02
TCGA-AA-3526	236	censored	0.8765855713486674	19.05
TCGA-AA-3986	235	censored	0.8765855713486674	19.05
TCGA-AA-3530	234	censored	0.8765855713486674	19.05
TCGA-NH-A5IV	233	censored	0.8765855713486674	19.32
TCGA-NH-A50V	232	censored	0.8765855713486674	19.32
TCGA-AH-6903	231	censored	0.8765855713486674	19.45
TCGA-G4-6317	230	relapsed	0.8727743297341081	19.45
TCGA-DY-A1DG	229	relapsed	0.8689630881195486	19.45
TCGA-AG-3909	228	censored	0.8689630881195486	19.97
TCGA-AA-3562	227	censored	0.8689630881195486	19.97
TCGA-AA-3560	226	censored	0.8689630881195486	19.97
TCGA-AG-3609	225	censored	0.8689630881195486	19.97
TCGA-AG-3612	224	censored	0.8689630881195486	19.97
TCGA-AG-3882	223	censored	0.8689630881195486	19.97
TCGA-AA-A01R	222	relapsed	0.8650488399748659	19.97
TCGA-CM-4744	221	censored	0.8650488399748659	20.01
TCGA-A6-6780	220	censored	0.8650488399748659	20.11
TCGA-A6-6782	219	censored	0.8650488399748659	20.27
TCGA-D5-6530	218	censored	0.8650488399748659	20.4
TCGA-A6-6650	217	censored	0.8650488399748659	20.6
TCGA-DY-A1DC	216	relapsed	0.8610439842342416	20.7
TCGA-EI-6508	215	censored	0.8610439842342416	20.89
TCGA-AA-3955	214	censored	0.8610439842342416	20.96
TCGA-AG-A002	213	censored	0.8610439842342416	20.96
TCGA-AG-3725	212	censored	0.8610439842342416	20.96
TCGA-AA-3549	211	censored	0.8610439842342416	20.99
TCGA-DC-4745	210	censored	0.8610439842342416	20.99
TCGA-AD-6965	209	relapsed	0.8569241565584796	21.29
TCGA-AD-6548	208	censored	0.8569241565584796	21.35
TCGA-AA-3968	207	censored	0.8569241565584796	21.98
TCGA-CK-5914	206	censored	0.8569241565584796	21.98
TCGA-AA-A00Z	205	censored	0.8569241565584796	21.98
TCGA-CM-5344	204	censored	0.8569241565584796	22.01
TCGA-A6-6138	203	censored	0.8569241565584796	22.5
TCGA-A6-6648	202	relapsed	0.8526819577636356	22.54
TCGA-AZ-4615	201	relapsed	0.8484397589687918	22.6
TCGA-AA-3556	200	censored	0.8484397589687918	23
TCGA-CM-4743	199	censored	0.8484397589687918	23.03
TCGA-D5-5537	198	relapsed	0.8441547096810705	23.72
TCGA-AA-3950	197	censored	0.8441547096810705	23.98
TCGA-AA-3941	196	censored	0.8441547096810705	23.98

TCGA-AA-3979	195	censored	0.8441547096810705	23.98
TCGA-AA-3553	194	censored	0.8441547096810705	23.98
TCGA-AA-3520	193	censored	0.8441547096810705	24.01
TCGA-A6-6140	192	censored	0.8441547096810705	24.11
TCGA-A6-6653	191	censored	0.8441547096810705	24.38
TCGA-AD-6890	190	censored	0.8441547096810705	24.51
TCGA-AA-3819	189	censored	0.8441547096810705	25
TCGA-CM-4747	188	censored	0.8441547096810705	25
TCGA-AG-3901	187	censored	0.8441547096810705	25
TCGA-AA-3977	186	censored	0.8441547096810705	25
TCGA-DC-6682	185	censored	0.8441547096810705	25.03
TCGA-DC-6683	184	censored	0.8441547096810705	25.03
TCGA-AA-3812	183	relapsed	0.8395418424150538	25.03
TCGA-DC-4749	182	censored	0.8395418424150538	25.03
TCGA-AA-A01P	181	relapsed	0.8349034896945287	25.03
TCGA-AD-6895	180	censored	0.8349034896945287	25.07
TCGA-D5-6533	179	censored	0.8349034896945287	25.46
TCGA-G4-6322	178	relapsed	0.830213020651301	25.92
TCGA-DC-6681	177	censored	0.830213020651301	25.95
TCGA-AG-A00H	176	relapsed	0.8254959012157822	25.95
TCGA-AA-3949	175	censored	0.8254959012157822	25.99
TCGA-AA-3976	174	censored	0.8254959012157822	25.99
TCGA-AA-3538	173	censored	0.8254959012157822	25.99
TCGA-AG-3999	172	relapsed	0.8206965064412718	25.99
TCGA-AG-A032	171	relapsed	0.8158971116667614	26.02
TCGA-CM-4748	170	censored	0.8158971116667614	26.02
TCGA-DC-5337	169	censored	0.8158971116667614	26.02
TCGA-G4-6588	168	censored	0.8158971116667614	26.15
TCGA-G4-6320	167	censored	0.8158971116667614	26.41
TCGA-AH-6897	166	censored	0.8158971116667614	26.41
TCGA-G5-6641	165	censored	0.8158971116667614	26.41
TCGA-AA-3710	164	censored	0.8158971116667614	26.97
TCGA-AA-3517	163	relapsed	0.8108916079142046	26.97
TCGA-AA-3994	162	censored	0.8108916079142046	27
TCGA-CM-4751	161	censored	0.8108916079142046	27
TCGA-AG-A023	160	relapsed	0.8058235353647408	27
TCGA-AA-3982	159	censored	0.8058235353647408	27
TCGA-AA-A00O	158	censored	0.8058235353647408	27
TCGA-A6-6137	157	censored	0.8058235353647408	27.07
TCGA-AU-6004	156	censored	0.8058235353647408	27.07
TCGA-AD-6963	155	censored	0.8058235353647408	27.4
TCGA-AF-3914	154	relapsed	0.8005909150052296	27.79
TCGA-G4-6304	153	relapsed	0.7953582946457183	28.22
TCGA-G4-6303	152	relapsed	0.790125674286207	28.65
TCGA-AA-3532	151	censored	0.790125674286207	28.98
TCGA-AA-3534	150	censored	0.790125674286207	28.98
TCGA-CM-6164	149	censored	0.790125674286207	29.01
TCGA-CM-5341	148	censored	0.790125674286207	29.04
TCGA-A6-5667	147	censored	0.790125674286207	29.14
TCGA-A6-5660	146	censored	0.790125674286207	29.17
TCGA-QG-A5YW	145	censored	0.790125674286207	29.43
TCGA-AA-3870	144	censored	0.790125674286207	29.96
TCGA-AF-4110	143	censored	0.790125674286207	29.96
TCGA-AG-3728	142	censored	0.790125674286207	29.96
TCGA-AA-A00E	141	censored	0.790125674286207	29.99
TCGA-AA-3862	140	censored	0.790125674286207	30.03
TCGA-AA-3861	139	censored	0.790125674286207	30.03
TCGA-CM-5349	138	censored	0.790125674286207	30.06
TCGA-A6-5659	137	censored	0.790125674286207	30.42
TCGA-AG-A01N	136	censored	0.790125674286207	30.98
TCGA-AA-3877	135	censored	0.790125674286207	30.98
TCGA-DC-5869	134	censored	0.790125674286207	30.98
TCGA-AA-A01I	133	censored	0.790125674286207	30.98
TCGA-AA-A01K	132	censored	0.790125674286207	30.98
TCGA-AA-3860	131	censored	0.790125674286207	31.04
TCGA-AA-3858	130	censored	0.790125674286207	31.04
TCGA-QG-A5Z2	129	censored	0.790125674286207	31.27
TCGA-A6-5657	128	censored	0.790125674286207	31.6
TCGA-A6-5666	127	relapsed	0.7839042122839533	31.87
TCGA-AG-3902	126	censored	0.7839042122839533	32
TCGA-AG-3578	125	censored	0.7839042122839533	32

TCGA-AA-A01F	124	censored	0.7839042122839533	32
TCGA-AA-3855	123	censored	0.7839042122839533	32.03
TCGA-A6-2684	122	relapsed	0.7774787679209701	32.06
TCGA-A6-4107	121	censored	0.7774787679209701	32.42
TCGA-A6-3809	120	censored	0.7774787679209701	32.72
TCGA-A6-5656	119	censored	0.7774787679209701	32.88
TCGA-QG-A5YX	118	censored	0.7774787679209701	32.95
TCGA-AG-3732	117	censored	0.7774787679209701	32.95
TCGA-G4-6627	116	relapsed	0.7707763647492377	32.95
TCGA-AY-5543	115	censored	0.7707763647492377	32.98
TCGA-AA-3947	114	censored	0.7707763647492377	32.98
TCGA-AA-3815	113	censored	0.7707763647492377	33.02
TCGA-AA-A02W	112	relapsed	0.7638944329211196	33.02
TCGA-AA-A01T	111	censored	0.7638944329211196	33.02
TCGA-AA-3851	110	censored	0.7638944329211196	33.05
TCGA-D5-5538	109	relapsed	0.7568862271145038	33.08
TCGA-A6-3808	108	censored	0.7568862271145038	33.31
TCGA-A6-5661	107	censored	0.7568862271145038	33.51
TCGA-AF-3911	106	relapsed	0.74974579100965	33.51
TCGA-F4-6808	105	censored	0.74974579100965	33.64
TCGA-AA-3548	104	censored	0.74974579100965	33.97
TCGA-AA-3956	103	censored	0.74974579100965	34
TCGA-AA-3531	102	censored	0.74974579100965	34
TCGA-AG-3593	101	censored	0.74974579100965	34
TCGA-AG-3592	100	censored	0.74974579100965	34
TCGA-AA-A00F	99	censored	0.74974579100965	34
TCGA-AA-3975	98	censored	0.74974579100965	34.03
TCGA-G5-6235	97	relapsed	0.7420164529580041	34.23
TCGA-AF-3400	96	censored	0.7420164529580041	34.46
TCGA-A6-3807	95	censored	0.7420164529580041	34.63
TCGA-AG-3887	94	relapsed	0.7341226609052595	34.95
TCGA-AA-A010	93	censored	0.7341226609052595	34.95
TCGA-AG-3893	92	censored	0.7341226609052595	34.99
TCGA-A6-2680	91	censored	0.7341226609052595	35.09
TCGA-F4-6463	90	censored	0.7341226609052595	35.71
TCGA-G4-6586	89	censored	0.7341226609052595	35.78
TCGA-AG-4001	88	censored	0.7341226609052595	36.01
TCGA-AA-3524	87	censored	0.7341226609052595	36.01
TCGA-AA-3854	86	censored	0.7341226609052595	36.01
TCGA-F5-6812	85	censored	0.7341226609052595	36.47
TCGA-A6-3810	84	censored	0.7341226609052595	36.5
TCGA-AA-3841	83	censored	0.7341226609052595	36.93
TCGA-CM-4746	82	censored	0.7341226609052595	36.99
TCGA-AG-A011	81	censored	0.7341226609052595	36.99
TCGA-AG-3731	80	censored	0.7341226609052595	36.99
TCGA-AA-A01Z	79	relapsed	0.7248299689950664	36.99
TCGA-AA-3522	78	censored	0.7248299689950664	37.02
TCGA-AA-3495	77	censored	0.7248299689950664	37.02
TCGA-AF-2689	76	relapsed	0.7152927325609207	37.25
TCGA-A6-2681	75	relapsed	0.7057554961267751	37.78
TCGA-AF-2693	74	censored	0.7057554961267751	37.94
TCGA-AA-A00L	73	censored	0.7057554961267751	38.01
TCGA-AA-A00A	72	censored	0.7057554961267751	38.01
TCGA-F5-6861	71	censored	0.7057554961267751	38.11
TCGA-AH-6544	70	censored	0.7057554961267751	38.53
TCGA-AA-3837	69	censored	0.7057554961267751	38.96
TCGA-G4-6311	68	censored	0.7057554961267751	39.39
TCGA-A6-2671	67	relapsed	0.6952218320054799	39.91
TCGA-AA-3972	66	relapsed	0.6846881678841847	39.95
TCGA-AG-3587	65	relapsed	0.6741545037628897	39.95
TCGA-AA-A02Y	64	censored	0.6741545037628897	39.95
TCGA-AA-A02F	63	censored	0.6741545037628897	39.95
TCGA-AF-A56K	62	relapsed	0.6632810440247785	39.98
TCGA-A6-2674	61	relapsed	0.6524075842866673	40.05
TCGA-AA-A03J	60	censored	0.6524075842866673	40.93
TCGA-AG-A02X	59	censored	0.6524075842866673	40.97
TCGA-AA-A00Q	58	censored	0.6524075842866673	41.98
TCGA-A6-2678	57	censored	0.6524075842866673	42.25
TCGA-QG-A5YV	56	censored	0.6524075842866673	42.74
TCGA-AF-2691	55	censored	0.6524075842866673	43
TCGA-F4-6807	54	censored	0.6524075842866673	43
TCGA-A6-2675	53	censored	0.6524075842866673	43.4

TCGA-A6-2675	53	censored	0.6524075842866673	43.4
TCGA-DC-6160	52	censored	0.6524075842866673	43.99
TCGA-G4-6306	51	censored	0.6524075842866673	44.65
TCGA-CI-6622	50	censored	0.6524075842866673	44.74
TCGA-A6-2679	49	censored	0.6524075842866673	44.88
TCGA-AG-4022	48	censored	0.6524075842866673	45.99
TCGA-A6-2672	47	censored	0.6524075842866673	46.62
TCGA-AA-3678	46	censored	0.6524075842866673	46.98
TCGA-CI-6623	45	censored	0.6524075842866673	47.4
TCGA-AG-3898	44	censored	0.6524075842866673	48
TCGA-CI-6624	43	censored	0.6524075842866673	48.16
TCGA-F5-6465	42	censored	0.6524075842866673	49.47
TCGA-AD-6889	41	relapsed	0.6364952041821145	49.8
TCGA-AG-A025	40	censored	0.6364952041821145	49.93
TCGA-AG-3598	39	censored	0.6364952041821145	50
TCGA-AA-3673	38	censored	0.6364952041821145	50
TCGA-CK-5913	37	censored	0.6364952041821145	51.28
TCGA-DC-6157	36	censored	0.6364952041821145	51.94
TCGA-AA-A029	35	censored	0.6364952041821145	51.94
TCGA-AA-3864	34	censored	0.6364952041821145	52.96
TCGA-AA-3664	33	censored	0.6364952041821145	53.98
TCGA-G4-6307	32	censored	0.6364952041821145	54.99
TCGA-A6-A56B	31	relapsed	0.6159631008214012	55.12
TCGA-D5-5541	30	censored	0.6159631008214012	55.88
TCGA-D5-5540	29	censored	0.6159631008214012	56.04
TCGA-AA-3506	28	censored	0.6159631008214012	57.98
TCGA-AZ-4315	27	censored	0.6159631008214012	58.34
TCGA-AA-3655	26	censored	0.6159631008214012	60.97
TCGA-G4-6315	25	censored	0.6159631008214012	61.86
TCGA-AG-A02N	24	censored	0.6159631008214012	61.93
TCGA-AA-3510	23	censored	0.6159631008214012	63.93
TCGA-AF-A56L	22	censored	0.6159631008214012	65.93
TCGA-AZ-6601	21	relapsed	0.5866315245918106	74.57
TCGA-AZ-4313	20	censored	0.5866315245918106	75.89
TCGA-G4-6297	19	relapsed	0.5557561811922416	78.65
TCGA-G4-6628	18	censored	0.5557561811922416	79.63
TCGA-CK-6747	17	censored	0.5557561811922416	82.88
TCGA-G4-6625	16	relapsed	0.5210214198677264	84.23
TCGA-AA-3697	15	censored	0.5210214198677264	84.99
TCGA-CK-4950	14	censored	0.5210214198677264	85.38
TCGA-AZ-5407	13	censored	0.5210214198677264	88.14
TCGA-DM-A28M	12	censored	0.5210214198677264	95.11
TCGA-AZ-4681	11	censored	0.5210214198677264	106.67
TCGA-AZ-4308	10	censored	0.5210214198677264	109.2
TCGA-AG-A036	9	censored	0.5210214198677264	117.02
TCGA-DM-A0X9	8	censored	0.5210214198677264	119.61
TCGA-DM-A28E	7	censored	0.5210214198677264	119.84
TCGA-CK-6751	6	censored	0.5210214198677264	124.18
TCGA-DY-A0XA	5	censored	0.5210214198677264	126.35
TCGA-DM-A1D0	4	censored	0.5210214198677264	130.55
TCGA-G4-6293	3	censored	0.5210214198677264	133.08
TCGA-DM-A282	2	censored	0.5210214198677264	139.06
TCGA-CK-4948	1	censored	0.5210214198677264	147.9

</pre></body></html>
